# Barriers to Wellness Among General Surgery Residents During the COVID-19 Pandemic: Qualitative Analysis of Survey Responses

**DOI:** 10.2196/72819

**Published:** 2025-11-24

**Authors:** Idil Bilgen, Matthew Castelo, Emma Reel, May-Anh Nguyen, Brittany Greene, Justin Lu, Savtaj Brar, Tulin Cil

**Affiliations:** 1 School of Medicine, Koç University, Istanbul, Turkey; 2 Department of Surgery, University of Toronto, Toronto, ON, Canada; 3 Institute of Health Policy, Management and Evaluation, Dalla Lana School of Public Health, University of Toronto, Toronto, ON, Canada; 4 Temerty Faculty of Medicine, University of Toronto, Toronto, ON, Canada; 5 Princess Margaret Cancer Centre, University Health Network, OPG Wing, 6th Floor, 5W278, 610 University Ave, Toronto, ON, M5G 2M9, Canada, 1 4169464501 ext 3984, 1 4169464429

**Keywords:** burnout, COVID-19, general surgery, internship and residency, qualitative

## Abstract

**Background:**

Health care provider burnout worsened during the COVID-19 pandemic.

**Objective:**

This qualitative study described general surgery residents’ perceptions of burnout and the impact of the COVID-19 pandemic and their attitudes toward wellness initiatives.

**Methods:**

General surgery residents at a large training program in Canada completed a 21-item survey focused on self-reported burnout, mental health, perceptions of wellness resources, and the effects of the COVID-19 pandemic. Free-text responses were extracted for qualitative thematic content analysis. A coding framework was established, and emergent themes were identified.

**Results:**

A total of 62% (51/82) of the residents completed the survey. Most respondents were senior residents (21/51, 41%) and identified as male (32/51, 63%). In total, 65% (33/51) of the residents met the criteria for burnout. Three themes were identified: (1) the culture of general surgery does not promote wellness, (2) the COVID-19 pandemic worsened existing access to vacation days and rest, and (3) wellness education in general surgery is ineffective and onerous to complete. General surgery residents emphasized the rigid lifestyle and culture of the specialty. Residents said that the idea of wellness was poorly executed. COVID-19 protocols increased the acceptance of taking sick days, but this was offset by staff shortages during the pandemic. Finally, residents emphasized the inefficacy of wellness education. They felt that they did not lack knowledge on reaching wellness but simply lacked the adequate time and resources to improve their well-being.

**Conclusions:**

There are persistent concerns within the culture of general surgery that were further impacted by workload and stress during the pandemic. These results may inform future programmatic efforts to decrease resident burnout.

## Introduction

Burnout is an occupational syndrome characterized by emotional exhaustion, detachment, poor self-worth, and low personal accomplishment [1]. It is common among medical professionals [2,3] and has been associated with worse patient care, increased medical errors, poor physician mental health, and training attrition [4-7]. A particularly vulnerable group of physicians is surgical trainees, as identified in several high-impact publications [2,8,9]. General surgery residents undergo an extended period of training (5 or more years) and are subjected to long work hours and chronic sleep deprivation. Indeed, these are among the reasons most often cited by trainees seriously considering leaving general surgery residency [10]. However, efforts to restrict duty hours have not clearly demonstrated benefit [11], and it has become clear that burnout in general surgery training is a complex phenomenon. Other factors such as discrimination and sexual harassment experienced during training may certainly contribute to both attrition and burnout [8].

The COVID-19 pandemic further exacerbated many of these preexisting issues while introducing new stressors to the training environment [12,13]. General surgery residents were called upon to assist in overwhelmed intensive care units (ICUs) during successive COVID-19 waves. Aside from missed surgical experience and disruption of surgical rotations, the stress of working with patients with COVID-19 was a contributing factor to physician burnout [14-18]. The negative effects of the pandemic have been reflected in the physical health, safety, and mental well-being of residents [19-25]. While there are emerging data on the impact of COVID-19 on resident wellness [12,13], there are minimal data on understanding residents’ experiences of the pandemic and their relationship with the culture of general surgery training more broadly. Given the larger systemic issues within training programs beyond work hours and redeployment, it is important to generate resident-specific data [26].

The aim of this study was to understand general surgery residents’ perceptions of burnout and the interaction with the COVID-19 pandemic experience through qualitative analysis of a wellness survey conducted at a large university-based training program.

## Methods

### Study Design 

This was a qualitative descriptive study [27,28] using free-text responses to a cross-sectional survey conducted within the General Surgery residency training program at the University of Toronto.

### Participants  

The General Surgery residency program at the University of Toronto is the largest such training program in Canada, comprising 84 clinical and research residents completing full-time graduate degrees [29]. The survey was administered through SurveyMonkey (SurveyMonkey Inc) to all registered general surgery residents as of February 16, 2021. Two additional email reminders were sent 2 weeks apart. Two residents who developed the survey were excluded (MAN and BG).  

### Study Team

The study team primarily responsible for designing the survey included SB (the program director of the General Surgery residency program at the University of Toronto), TC (the wellness lead and active researcher in surgical education), MAN (general surgery senior resident), and BG (general surgery senior resident). IB and ER conducted the qualitative analysis and thematic coding. ER is an experienced social worker with expertise in qualitative methodology. MC is a surgeon and clinical epidemiologist who conducted the statistical analysis.

### Survey Development and Content

The original objectives of the survey were to assess resident burnout, self-reported mental health, and perceptions of available wellness and support resources in response to the COVID-19 pandemic. These results have been reported separately [30]. At the time of survey administration, Ontario was experiencing ongoing COVID-19–related disruption with widespread public health restrictions. The period immediately before the survey (December 2020 and January 2021) was marked by a wave of SARS-CoV-2 infections, high ICU use, and residency training interruption and redeployment [31]. This directly led to the development of this survey.

The 21 survey questions were developed and reviewed for clarity and sensibility. The entire survey is shown in Multimedia Appendix 1. It consisted of questions reporting demographics, level of training, perceived importance of wellness, the efficacy of wellness initiatives undertaken by the residency program, the availability of such resources, and regret for pursuing surgical residency. We also measured self-identified burnout, formal burnout measures, and mental health concerns. The survey was transferred to an online format [32] and pilot-tested before administration. A total of 81 free-text responses were available for 17 survey questions, which underwent coding and formed the data source for this qualitative analysis.

Burnout was formally assessed using the Maslach Burnout Inventory–Human Services Survey for Medical Personnel (MBI-HSS) [1]. The MBI-HSS is a validated psychometric tool created to study burnout in medical professionals and assesses 3 dimensions of burnout: emotional exhaustion, depersonalization, and personal accomplishment (Multimedia Appendix 1). Higher scores for emotional exhaustion and depersonalization, along with lower scores for personal accomplishment, indicate higher degrees of burnout. The presence of burnout according to the MBI-HSS was defined in accordance with multiple high-impact studies [3,8,33,34]. Participants were considered to have burnout if they had at least weekly symptoms on a subset of 6 scale items that form the abbreviated MBI-HSS [35].

### Analysis

Participant characteristics were tabulated and analyzed using descriptive statistics. The survey questions and corresponding free-text responses were extracted for qualitative thematic content analysis [36]. Comments were coded into specific themes. An inductive and semantic qualitative approach was undertaken to obtain a thorough grasp of the residents’ perceptions of wellness and burnout in general surgery during the pandemic [27,28,37]. A reflexivity practice was embedded in the analysis process. Analysts engaged in memoing while independently reviewing the data; these notes were discussed alongside the data as part of peer debriefing, during which themes and analyses were discussed within the study team and a coding framework was established based on consensus [27,28,36]. Furthermore, the analysts reflected on their positions within the research and how their own educational and professional identities may impact their interpretation of the data [38]. A primary analyst coded all the transcripts, with a second analyst coding a subset independently using Microsoft Excel (IB and ER).

### Ethical Considerations

The Research Ethics Board at the University of Toronto reviewed this study and granted ethics approval (study #40135). Informed consent was obtained from all participants, and they were given an opportunity to opt out at any time. Their participation in this study was not mandatory. The data were deidentified, and no compensation was provided.

## Results

### Participant Demographics and MBI-HSS

The survey was distributed to 82 general surgery residents at the University of Toronto, and 51 (62%) completed it. Demographics are presented in Table 1. Most respondents were senior residents (21/51, 41%), followed by junior residents (18/51, 35%) and research residents (11/51, 22%). Most respondents identified as male (32/51, 63%). Of note, the gender breakdown of the program at the time of the survey was 60% male. There was a nonsignificant increase in residents identifying themselves as having burnout during the pandemic compared to before the pandemic (32/51, 63% vs 29/51, 57%, respectively; *P*=.21). Most respondents (29/51, 57%) never or rarely regretted training in general surgery, whereas 37% (19/51) sometimes regretted it and only 6% (3/51) usually regretted it.

**Table 1.** Participant demographics (N=51).

Characteristic	Participants, n (%)
Year of training	
PGY^a^ 1	6 (12)
PGY 2	12 (24)
PGY 3	9 (18)
PGY 4	5 (10)
PGY 5	7 (14)
Research	11 (22)
Preferred not to answer	1 (2)
Level of training	
Junior resident	18 (35)
Senior resident	21 (41)
Research	11 (22)
Preferred not to answer	1 (2)
Self-identified gender	
Female	19 (37)
Male	32 (63)
Self-described burnout before the pandemic	
Maybe	7 (14)
No	15 (29)
Yes	29 (57)
Self-described burnout during the pandemic	
Maybe	10 (20)
No	9 (18)
Yes	32 (63)
Regret for general surgery residency	
Never	15 (29)
Rarely	14 (27)
Sometimes	19 (37)
Usually	3 (6)

a PGY: postgraduate year.

MBI-HSS subscale scores are presented in Table 2. They indicated that personal achievement occurred between once per week and a few times per week (median 4.62/7, IQR 4.00-5.31), emotional exhaustion occurred between once per month and a few times per month (median 2.67/7, IQR 1.94-3.44), and depersonalization occurred between a few times per year and once per month (median 1.80/7, IQR 1.20-2.50). A total of 65% (33/51) of the residents met the criteria for burnout using the MBI-HSS.

**Table 2.** Participant burnout scores (N=51).

	Values
Met criteria for burnout, n (%)	33 (65)
MBI-HSS^a^—emotional exhaustion score (range 0-6), median (IQR)	2.67 (1.94-3.44)
MBI-HSS—personal achievement score (range 0-6), median (IQR)	4.62 (4.00-5.31)
MBI-HSS—depersonalization score (range 0-6), median (IQR)	1.80 (1.20-2.50)

a MBI-HSS: Maslach Burnout Inventory–Human Services Survey for Medical Personnel.

### Emergent Themes

#### Overview

Our analysis identified 3 emergent themes in the free-text survey responses (Table 3). These were (1) the culture of general surgery does not promote wellness, (2) the COVID-19 pandemic worsened access to time off, and (3) wellness education in general surgery is ineffective and onerous to complete. These themes are presented with supporting quotes in the following sections.

**Table 3.** Emergent themes and selected quotes.

Theme	Selected quotes
The culture of general surgery does not promote wellness	“I am not aware of wellness resources available specifically from the General Surgery department.” [Participant ID 35] “Residents were left out of the picture on many occasions. Mass emails to the resident body not very helpful.” [Participant ID 25] “Wellness depends on having free time. Ie time to speak to a psychologist, see a doctor, rest, engage with family etc. Wellness curriculum/didactic teaching is further inhibitory to resident access to the above activities (especially much needed appointments) by further scheduling activities into already full schedules.” [Participant ID 7]
The COVID-19 pandemic worsened existing access to vacation days and rest	“Pre-COVID times—came in while still sick out of perceived expectation.” [Participant ID 34] “...never even bothered to try—rumors about how rarely they were approved, plus seeing people already doing at—or over—PARO max call.” [Participant ID 19] “I was discouraged from taking lieu days early in my postgraduate training.” [Participant ID 23]
Wellness education in general surgery is ineffective and onerous to complete	“...any format with flexibility—forced wellness activities feel counter-intuitive.” [Participant ID 19] “Give residents protected time away from hospital/lecture hall in casual/relaxed setting. Don’t educate re: wellness; facilitate wellness.” [Participant ID 22] “As residents, we have little to no control over our schedules, or our life in general. Wellness education and initiatives need to come from the top down and be incorporated within the residency program. For example, taking a lieu day should not be something we necessarily ask for, however, when a chief resident schedules a resident to be on call on a holiday, they should subsequently ask when they want to take their lieu day. This type of approach would ensure that the resident feels comfortable taking the lieu day, and remove any sense of guilt they may feel taking a lieu day. When it comes to wellness education, I think the goals are two-fold. 1. Education at the higher levels so that individuals in positions of power, eg, Staff, Fellows, Chief Residents, Sr. Residents, are educated on how to create an environment and workplace that promotes wellness for everyone involved. 2. Individual level education on how we can each individually engage in activities to promote our wellness.” [Participant ID 32] “Increasing the number of the residents in the program might help decrease the stress and load of work on the residents and number of calls.” [Participant ID 43]

#### The Culture of General Surgery Does Not Promote Wellness

General surgery residents emphasized the rigid lifestyle and culture of their chosen specialty. Multiple residents said that the idea of wellness existed only theoretically and was not well executed in reality. Residents admitted that achieving wellness appeared unlikely due to the perceived expectations of general surgery residency, claiming that *“*true wellness is somewhat contradictory to the classic culture of surgical residency*”* (participant 23):

*I’m not sure how much wellness initiatives targeted at residents specifically will help promote their wellness while the culture of surgery and residency remains what it is. This is a very difficult and complex issue to address.*

[Participant 23]

In terms of suggested changes, residents reported that feeling supported by hospital coworkers (eg, staff surgeons, allied health professionals, and administrators) would provide them with a greater sense of security. They felt that achieving wellness requires respect from hospital staff, improvements in the work environment, and a deeper appreciation of efforts and personal sacrifices:

*Wellness comes from perceiving high self-worth and that our work environment finds us valuable. If I’m treated with respect and valued at work that will generate resilience against burn out... Wellness comes from the program/work environment address[ing] that concern and changing for the better. No podcast/retreat will ever address those concerns adequately.*

[Participant 8]

One aspect of surgical culture that was raised was the lack of time to focus on wellness; the perception was that didactic or educational interventions (eg, mandatory retreats) were at times contradictory to personal efforts at wellness. Residents acknowledged that greater recognition and valuation of this need could improve wellness:

*No more lectures, no more retreats. No more anything that already takes away from our precious little spare time to be human. Encourage and foster and promote more spare time away from work/study to be human and do things that let us recharge. ...If resident wellness programs actually wanted to promote wellness, they would target the systematic issues that makes residency sometimes needlessly grueling as opposed to further taking away our time.*

[Participant 16]

Despite these challenges, one resident did feel that it was “reassuring that the General Surgery program has a Wellness Lead and is working towards improving wellness...” [participant 32]*.* Specific mentors were highlighted as being supportive, particularly the program directors.

In particular, staff mentors were identified as being important resources during the pandemic, with specific staff surgeons being named. However, one resident noted that they were “left out of the picture on many occasions” (participant 25). This may suggest that the program’s response to COVID-19 worsened resident involvement and dissemination of information.

#### The COVID-19 Pandemic Worsened Existing Access to Time Off

Residents highlighted challenges in access to vacation and lieu days during the COVID-19 pandemic. As discussed previously, they emphasized the lack of adequate time for self-care due to long work hours and felt at times undervalued. Interestingly, the pandemic increased the perceived acceptance of taking sick days, likely due to strict COVID-19 protocols implemented at hospitals and training sites. Before these changes, participants reported that it was rare to use sick time:

...*pre-COVID times—came in while still sick out of perceived expectation*.

[Participant 19]

*I had never taken a sick day until during COVID, when I had a fever for a few days and had to wait for my test result to come back. Before then, I have certainly worked while sick and wished I was at home-but never asked to leave/stay home. Unsure what the response would have been if I had asked to go home or not to come in because I wasn’t feeling well. It actually felt nice during COVID to be forced to stay home until my test result came back. I got to rest and recover instead of just powering through...*

[Participant 33]

However, this was offset by cancelled vacation days as a result of COVID-19 health care personnel shortages. While residents are entitled to an annual 4 weeks of vacation time, some participants noted that they “only took 2 weeks of vacation due to COVID scheduling” (participant 18):

*Lieu days quite difficult to take due to clinical demands... Have never taken an educational day in residency. Took 2/4 weeks’ vacation (rest was cancelled due to pandemic).*

[Participant 40]

In addition to sick days and vacation days, lieu days were also discussed. As per the employment contracts relevant to the study population, residents are eligible for a paid day off if required to work on a recognized holiday to be taken within 90 days of the holiday [39]. Participants admitted that they felt that lieu days only existed theoretically—there was a perceived expectation not to use the paid day off as doing so would worsen existing coverage shortages and would be unfair to coresidents. Some went as far as saying that they were “discouraged to take [lieu days]” (participant 44) and that “it is not acceptable in my residency culture” (participant 41):

*I have very rarely seen anyone in [the] general surgery program (other than off service) actually use a lieu day. Given how short residents we are at most sites, taking lieu days feels like it will penalize the other residents, who will have to cover.*

[Participant 35]

Suggestions offered by participants included streamlining requests for vacation time so that they were centralized and coordinated, encouraging residents to plan lieu days when scheduled to work holidays, and avoiding ward rounds before scheduled teaching activities:

*When a chief resident schedules a resident to be on call on a holiday, they should subsequently ask when they want to take their lieu day.*

[Participant 32]

#### Wellness Education in General Surgery Is Ineffective and Onerous to Complete

Residents discussed the inefficacy of wellness education. There were differences in how residents perceived wellness and how it was conveyed by the residency program. While many initiatives focused on wellness education or skills, study participants emphasized that they preferred more free time rather than “wellness through mandatory modules and activities” (participant 35):

*Promoting wellness starts with valuing residents’ time. Improving service to education ratio would allow us to focus more on education, and it would reduce our overall hours spent in hospital, which in turn could open up the opportunity for wellness related activities.*

[Participant 35]

Participants were not enthusiastic about wellness programs and, instead, preferred time for self-care. It was noted that scheduling personal health appointments (including counseling) was challenging, and many residents completed these activities on postcall days:

*Wellness depends on having free time, ie, time to speak to a psychologist, see a doctor, rest, engage with family etc. Wellness curriculum/ didactic teaching is further inhibitory to resident access to the above activities (especially much needed appointments) by further scheduling activities into already full schedules.*

[Participant 7]

On the basis of their free-text responses, residents felt that they did not lack knowledge on reaching wellness but simply lacked the adequate time and resources for carrying out wellness-promoting behaviors. For this reason, many were against mandatory or scheduled educational sessions as they did not attempt to address the “root causes of unwellness” (participant 24):

*The ultimate source of burnout is the work burden and not the lack of knowledge on how to be well. We require time to be well. It would be useful if we had academic half days that are dedicated for resident wellness (time for us to make and attend appointments when we are not post call, counseling sessions, catch up on life in general).*

[Participant 18]

However, not all wellness-related activities were considered ineffective. Social events held by the program that encouraged socializing with coresidents were praised, although the challenges posed by COVID-19 regarding these types of events were acknowledged. One resident encouraged virtual events instead:

*Program can facilitate a virtual social like a cooking class or trivia or other ways to engage residents on a program level.*

[Participant 51]

Rather than wellness education aimed at residents, some suggested training that could “focus on staff understanding [of] resident wellness” (participant 44)*.* Finally, some participants suggested that wellness activities could take the form of more concrete actions toward clinical stressors such as unnecessary consultations and pages and “respect[ing] academic half days (no rounding before teaching)” (participant 39). One resident noted that call requirements could be lightened to improve wellness:

*Doing one weekend call/month would be ideal for wellness.*

[Participant 43]

## Discussion

This cross-sectional qualitative study of free-text responses among general surgery residents surveyed about burnout and wellness initiatives during the COVID-19 pandemic revealed substantial concerns regarding surgical culture, availability of free time, and the perception of wellness initiatives. Residents identified interconnected issues that resulted in little free time to focus on their well-being, and these problems were exacerbated by mandatory modules and educational sessions that were not perceived as helpful and further impeded time for rest.

Surgery has long been recognized as having a rigid culture with heavy workload, long hours, high call demands, and complex patients [40]. There has been increasing recognition of these issues, but solutions are unclear. A major focus of research and policy has been on duty hour restrictions, which have been widely used in the United States and Europe but less so in Canada. These initiatives were spurred by concerns about patient safety and resident wellness over 20 years ago [41]. With time, evidence has accumulated suggesting that duty hour restrictions do not have a strong impact on resident wellness in surgery [11,42]. Instead, as the results of our study indicate, there are broader problems within surgical culture that contribute to burnout. In 2018, Hu et al [8] conducted a cross-sectional survey of the American general surgery resident population (7409 trainees), with 32% reporting gender-based discrimination, 17% reporting racial-based discrimination, and 30% reporting verbal or physical abuse during training. While our participants did not raise concerns about mistreatment or abuse directly, they pointed to systemic issues such as feeling undervalued and unsupported. Evidence has pointed to deep-rooted cultural issues within the surgical environment that contribute to bullying and burnout. These include hierarchical power structures and problematic individual surgeons with narcissistic tendencies that support these dynamics [43,44]. In a comprehensive examination of this issue, Munro and Phillips [45] quote evidence that surgical trainees are 3 times more likely to experience bullying compared to other trainees. Reasons again include the essential power imbalance that comes from a hierarchical work environment, the public nature of feedback in the operating room, and emulation of bullying behavior itself as a consequence of prolonged training years.

Our study demonstrated that, during the COVID-19 pandemic, some of the barriers to wellness were exacerbated. Although the survey was conducted during the pandemic and residents had experienced at least 2 substantial waves with increased pressures on hospitals and redeployments, participants did not directly relate these events to burnout. Rather, they raised concerns surrounding the cancellation of vacation time and identified issues with receiving entitled lieu days. Literature from early in the pandemic has shown a substantial impact on residents’ educational opportunities and well-being [12-14]. It is possible that our participants were affected by recall bias or they intentionally chose to raise broader issues with respect to burnout rather than focusing on the pandemic specifically. Furthermore, junior residents would have had limited to no prepandemic experience with residency training, thereby not having a comparative experience outside of the COVID-19 pandemic years. Finally, some stressors that are cited in this early pandemic literature may have faded, including poor access to personal protective equipment and fear of infection. In addition to vaccination providing robust protection from serious complications of COVID-19, our resident population was relatively young and healthy and could be expected to experience mild illness. This is an important finding as programs may need to shift away from wellness initiatives directed at pandemic stressors back to preexisting systemic issues in surgery.

There is literature showing that both individual and structural wellness interventions can reduce burnout among physicians [26]. Most of these take the form of mindfulness or stress reduction sessions and small group discussions. Our results suggest that these interventions would be poorly perceived by surgical residents, who felt that these further placed time demands on busy residents and may worsen burnout. Randomized studies of structural and organizational interventions are less common [11,46-48], but these were more widely supported by our participants and may have more success in this population. In a landmark study published in *The New England Journal of Medicine*, Bilimoria et al [11] randomized 117 general surgery residency programs in the United States to follow strict duty hour restrictions or a more flexible policy that waived rules on shift length and time between shifts. Residents working under the flexible policy were less likely to report patient safety concerns or negative effects on professionalism and education but noted a greater impact on their personal activities. Overall, the trial questioned the evidence base behind restrictive duty hour policies. In a similar trial conducted in Canada, ICU residents were randomized to overnight shifts between 12 and 24 hours in length [48]. Somatic symptoms among residents were more common in the 24-hour shift group, but rates of burnout were similar. There was a tendency toward more preventable errors in patient care occurring in the 12-hour shift group. This literature highlights tension between patient safety and resident wellness and, similar to our results, suggests that institutional policy may be less effective than broader cultural change and sense of belonging at improving burnout [49]. Further research as to the optimal ways to address burnout among trainees is critically needed.

This study has several strengths. Our response rate was high, indicating engagement with the residency program. We used formal qualitative analysis to explore the free-text responses to our survey rather than simple quantitative methods [36,50]. Our assessment of burnout was complemented by formal measures of burnout (MBI-HSS scores), and we have previously described these results [30]. Although data resulting from in-depth interviews may have been richer, given the limitations of conducting research during the pandemic, we were still able to secure a detailed set of responses from a relatively large group of general surgery residents who had experienced considerable disruption in training due to COVID-19. Our study is an important contribution to our understanding of barriers to improving wellness among general surgery residents.

This study has limitations. While the survey was distributed to 82 residents in the program, 51 (62%) completed it, and not all participants chose to offer free-text responses. Thus, our study cannot be considered a random sample of residents in the program. Those with particularly negative or strong opinions or experiences may have been more likely to use the free-text responses and have their views represented in this study. However, we found remarkable consistency in the responses, and the issues raised were not unexpected. As discussed previously, survey responses do not constitute as detailed a data source as interviews [51], although qualitative analyses of surveys are common. This approach is supported by the literature and the methodology of other similar studies, with free-text answers shown to be a rich source of data [52-55]. Inductive analysis is data-driven and an efficient method [37]. Our methodology maps closely to the recommendations of Thomas [56], including a close reading of text, creation of categories and themes, overlapping coding and revisiting uncoded text, and continuing revision and refinement of the category system. We also conducted consistency checks, such as independent parallel coding, checking on the clarity of categories, and research team discussions [56]. However, we still plan to conduct in-depth telephone interviews to further explore the findings generated by this study. We did not provide participants with formal definitions for terms such as “wellness” or “wellness education.” Thus, there may have been heterogeneity in how participants understood these core concepts. These data, particularly with respect to COVID-19, may not be generalizable to other programs. At the time the survey was completed, Toronto had greater experience with COVID-19 in health care settings than other Canadian cities, and the residents likely had more disruption to their training compared to colleagues in less dense centers. Conversely, they were likely less disrupted compared to American programs in similarly large cities—Canada had far less COVID-19–related morbidity on a per capita basis compared to the United States [57]. The residency program at the University of Toronto is also highly academic, with a larger number of research residents and unique work environments that may further limit generalizability to other institutions. A final limitation of our analysis is that we did not conduct member checking, where the conclusions of the study are fed back to the respondents to ensure validity [58].

This qualitative analysis of survey responses among general surgery residents working during the COVID-19 pandemic led to the recognition of 3 important themes related to wellness: the friction between the culture of surgery and wellness prioritization, the impact of the pandemic on working hours, and the difficulties with wellness interventions. These results represent the resident experience at a unique time in health care and may inform future efforts to design interventions to decrease burnout.

## Supplementary material

10.2196/72819 Multimedia Appendix 1 General surgery residency wellness and burnout survey.

## Abbreviations

ICU: intensive care unit
MBI-HSS: Maslach Burnout Inventory–Human Services Survey for Medical Personnel
