# Supplementary material for: Barriers to Wellness Among General Surgery Residents During the COVID-19 Pandemic: Qualitative Analysis of Survey Responses
Source: JMIR Perioper Med. 2025 Nov 24;8:e72819. doi: 10.2196/72819 (PMC12643399; doi:10.2196/72819)

**Supplementary Material 1.** General Surgery Residency Wellness and Burnout Survey.

| **Question Number** | **Question** | **Possible responses** |
| --- | --- | --- |
| 1 | Year of Study | PGY1  PGY2  PGY3  PGY4  PGY5  Research  Prefer not to answer |
| 2 | Gender | Female  Male  Other (free text response)  Prefer not to answer |
| 3 | What is your perception of the importance placed on resident wellness in relation to other required aspects of your program? | A priority for the residency program  Somewhat a priority  Not a priority  I don’t know  Prefer not to answer |
| 4 | How effective are the wellness initiatives in your program? | Very effective  Effective  Somewhat effective  Not effective  I don’t know what wellness initiatives we have  Other (free text response)  Prefer not to answer |
| 5 | How effective are the wellness resources available to you in your program? | Very effective  Effective  Somewhat effective  Not effective  I don’t know what wellness resources we have  Other (free text response)  Prefer not to answer |
| 6 | Have you ever used the following wellness resources? (Tick all that apply) | Senior/Chief residents  Site Director  Program Director  General Surgery Wellness Lead  Postgraduate Medical Education Wellness Office  Professional Association of Residents in Ontario (PARO)  Ontario Medical Association Physician Health Program  Other (free text response)  Prefer not to answer |
| 7 | During the COVID-19 pandemic, which, if any, of the following wellness resources were helpful to you? Tick all that apply. | Senior/Chief residents  Site Director  Program Director  General Surgery Wellness Lead  Postgraduate Medical Education Wellness Office  Professional Association of Residents in Ontario (PARO)  Ontario Medical Association Physician Health Program  Other (free text response)  Prefer not to answer |
| 8 | During your residency training, have you personally experienced any of the following issues? (Select all that apply) | Depression  Suicide Attempt  Drug Use  Binge Drinking  Eating Disorder  I don’t know  Other (free text response)  Prefer not to answer |
| 9 | During the COVID-19 pandemic, did you experience any of the following issues? | Depression  Suicide Attempt  Drug Use  Binge Drinking  Eating Disorder  I don’t know  Other (free text response)  Prefer not to answer |
| 10 | The Professional Association of Residents of Ontario stipulates that every resident is entitled to 4 weeks of paid vacation each year in addition to 7 working days for educational purposes, a floating holiday, lieu day for working on statutory holidays, and 5 consecutive days during the winter holiday season. Check all that you have taken in the preceding academic year (July 1, 2019 – June 30, 2020) | 4 full weeks’ vacation  5 consecutive days of holiday vacation  Floating holiday  Lieu day  7 full educational days  Other (free text response)  Prefer not to answer |
| 11 | If applicable, thinking back to earlier academic years, check all that you have taken. | 4 full weeks’ vacation  5 consecutive days of holiday vacation  Floating holiday  Lieu day  7 full educational days  Other (free text response)  Prefer not to answer |
| 12 | During the COVID-19 pandemic, did you take your entitled leave? | Yes  No (free text response)  Prefer not to answer |
| 13 | During residency, have you ever taken sick days? | Yes  No, I have never required them  No, I was unable to  Other (free text response)  Prefer not to answer |
| 14 | During residency, have you ever taken lieu days? | Yes  No  Other (free text response)  Prefer not to answer |
| 15 | Prior to the Pandemic, how often did you engage in activities that help maintain wellness in your life each month? (10+, 5-10, less than 5, once, never) | Exercise  Napping  Meditation  Cooking  Social activities with fellow residents  Social activities with family members  Social activities with other groups  Talking to family  Talking to friends  Watching TV  Drinking alcohol  Hobbies  Reading for fun  Play an instrument  Attend a religious/spiritual activity  Tobacco  Counselling  Coaching  Other (free text response)  Prefer not to answer |
| 16 | Since the start of the Pandemic, how often do you engage in activities that help maintain wellness in your life each month? (10+, 5-10, less than 5, once, never) | Exercise  Napping  Meditation  Cooking  Social activities with fellow residents  Social activities with family members  Social activities with other groups  Talking to family  Talking to friends  Watching TV  Drinking alcohol  Hobbies  Reading for fun  Play an instrument  Attend a religious/spiritual activity  Tobacco  Counselling  Coaching  Other (free text response)  Prefer not to answer |
| 17 | Did you ever previously (prior to the Pandemic) perceive yourself as experiencing burnout? | Yes  Maybe  No |
| 18 | Do you currently (since the Pandemic) perceive yourself as experiencing burnout? | Yes  Maybe  No |
| 19 | How do you feel wellness education in residency training should be promoted? | Didactics  Off-site workshops  Online modules/videos  Podcasts  Program retreats  Team building exercises  Other (free text response)  Prefer not to answer |
| 20 | Given wellness and burnout issues, how often do you regret doing general surgery residency? | Never  Rarely  Sometimes  Often  Other (free text response)  Prefer not to answer |
| 21 | Do you have any further comments? | Yes (open free text)  No |

**Supplementary Material 2**. Sample of Maslach Burnout Inventory Human Services Survey for Medical Personnel.


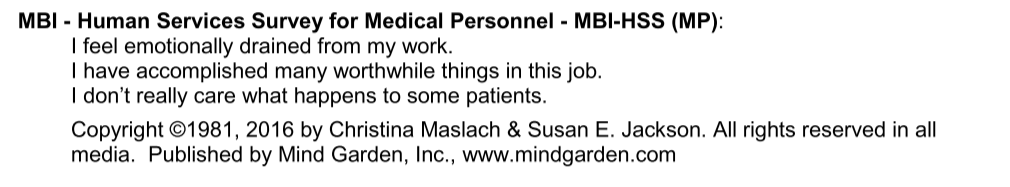

Supplement: Multimedia Appendix 1 [file periop-v8-e72819-s001.docx]
